# Optimising epilepsy care throughout the Afghan refugee crisis

**DOI:** 10.1016/S0140-6736(21)02224-8

**Published:** 2021-10-28

**Authors:** Arjune Sen, Asma Hallab, J Helen Cross, Josemir W Sander, Charles R Newton

**Affiliations:** Oxford Epilepsy Research Group, NIHR Biomedical Research Centre, Nuffield Department of Clinical Neurosciences, John Radcliffe Hospital, Oxford OX3 9DU, UK; Department of Psychiatry and Psychotherapy, Charité–Universitätsmedizin Berlin, corporate member of Freie Universität Berlin and Humboldt-Universität zu Berlin, Berlin, Germany; Developmental Neurosciences, UCL NIHR Biomedical Research Centre Great Ormond Street Institute of Child Health, London, UK; Department of Clinical and Experimental Epilepsy, UCL Queen Square Institute of Neurology, London, UK; Stichting Epilepsie Instellingen Nederland, Heemstede, Netherlands; Oxford Epilepsy Research Group, NIHR Biomedical Research Centre, Nuffield Department of Clinical Neurosciences, John Radcliffe Hospital, Oxford OX3 9DU, UK; Department of Psychiatry, University of Oxford, Oxford, UK; KEMRI–Wellcome Trust Research Programme, Centre for Geographic Medicine Research—Coast, Kilifi, Kenya

The takeover of Afghanistan by the Taliban in August, 2021, and the associated collapse of governmental institutions has led to innumerable security and humanitarian concerns, particularly for women and girls.^1^ Even before this event, more than 550 000 Afghans had been internally displaced during 2021.^2^ At the end of 2020, 3 million Afghans were internally displaced, and 2·6 million Afghan people were recognised as refugees or asylum seekers; most of them hosted in neighbouring Pakistan and Iran.^2^ The hastily arranged mass exodus, as international service personnel withdrew, has further spread Afghan refugees worldwide.

Although many people who were forced to leave Afghanistan are capable, literate, and determined, adjusting to new societies and lifestyles is not straightforward. Forcibly displaced people, within or outside Afghanistan, often have restricted access to security, shelter, and health-care facilities and inadequate nutrition. Some individuals are also at high risk of persecution and death.^1^

Among people living in refugee camps, the most reported neurological condition is epilepsy.^3^ Despite affecting over 50 million people worldwide and being treatable with affordable antiseizure medications in most people,^4^ epilepsy is a deeply stigmatised condition. This stigma is partly attributable to long-standing misconceptions about the neurological basis of seizures and other traditional beliefs, for example, that epilepsy is contagious. Convulsions are also unpredictable and difficult to witness, leading to fear and anxiety, including in health-care workers helping people who have been displaced.^5^ Asylum seekers can, therefore, be wary of declaring an epilepsy diagnosis, fearing that this will prejudice refugee status applications.^5^ All of these factors, coupled with, for example, a scarcity of medically trained personnel with appropriate expertise, contribute to forcibly displaced people with epilepsy receiving suboptimal care.

As the turmoil in Afghanistan continues to unfold, health-care systems should adapt to the increasing needs of forcibly displaced populations. Health-care workers should be trained in the essential management of seizures to help people in the acute setting. Improved knowledge about epilepsy, encouraging improved adherence to antiseizure medications, and destigmatisation will help improve prognosis. A transcultural approach seems crucial; for example, Afghan refugees and asylum seekers have previously reported feeling isolated in their contact with host–country institutions that, superficially, seem culturally similar.^5^ Awareness of common causes of epilepsy in Afghanistan, differential diagnoses for seizures, and the context that people are leaving, with special attention to hardships faced by women and girls, should help forcibly displaced Afghans with epilepsy and people who develop seizures to integrate into their receiving countries.
